# Author Correction: Structure and interactions of the endogenous human Commander complex

**DOI:** 10.1038/s41594-024-01273-y

**Published:** 2024-03-19

**Authors:** Saara Laulumaa, Esa-Pekka Kumpula, Juha T. Huiskonen, Markku Varjosalo

**Affiliations:** Institute of Biotechnology, Helsinki Institute of Life Science HiLIFE, University of Helsinki, Helsinki, Finland

**Keywords:** Cryoelectron microscopy, Protein-protein interaction networks

Correction to: *Nature Structural & Molecular Biology* 10.1038/s41594-024-01246-1, published online 8 March 2024.

In the version of the article initially published, the PDB accession codes were incorrect in Table 1 and the Data availability section, and have now been corrected to 8P0V, 8P0W and 8P0X in the HTML and PDF versions of the article.

