# Supplementary material for: Structure and interactions of the endogenous human Commander complex
Source: Nat Struct Mol Biol. 2024 Mar 8;31(6):925–38. doi: 10.1038/s41594-024-01246-1 (PMC11189303; doi:10.1038/s41594-024-01246-1)
Supplement: Supplementary file 1 — Supplementary Information [file 41594_2024_1246_MOESM1_ESM.pdf]

nature structural & molecular biology

Article

https://doi.org/10.1038/s41594-024-01246-1

Structure and interactions of the endogenous human Commander complex

In the format provided by the authors and unedited

Structure and Interactions of the Endogenous Human Commander Complex

Saara Laulumaa[1]†, Esa-Pekka Kumpula[1]†, Juha T. Huiskonen[1]*, Markku Varjosalo[1]*

[1]Institute of Biotechnology, Helsinki Institute of Life Science HiLIFE; 00014 University of Helsinki, Helsinki, Finland.

*Corresponding authors: Juha Huiskonen (juha.huiskonen@helsinki.fi) and Markku Varjosalo (markku.varjosalo@helsinki.fi)

†These authors contributed equally to this work.

Supplementary text

Model building

AP-MS indicated that the Commander complex contains a single copy of each subunit (**Fig. 1d**). We used AlphaFold2 (AF2) with a single copy of each sequence to generate an initial atomic model for the entire COMMD ring and different sub-complexes of the complete Commander complex. The COMMD proteins contain two domains: a C-terminal COMM domain unique to this family, and (except for COMMD6) a globular α-helical N-terminal domain (NTD). We predicted the structure of the hetero-decameric COMMD-ring without the N-terminal domains (NTDs), as it had the highest resolution that would allow identification of individual COMMD components in the density. AF2 predictions converged on a ring-like structure with varying orders of COMMD subunits. We used COMMD6 to anchor the models to the cryo-EM density and determined the correct order of COMMD subunits in the ring by density fit. We used the best fitting model within the COMMD domains as a starting point and refined the model into density (**Fig. 3a**). To validate our assignment of different COMMD subunits, we tested placements of all COMMD proteins against all possible sites in the density. Ranking of these placements by model-to-map cross-correlation after real-space refinement showed that our initial assignment was ranked highest (**Fig. 3b-d**).

We next built models into the remaining areas of the cryo-EM density. The predicted N-terminal NN-CH domain of CCDC93 fits well into the density on the side of the COMMD-ring between NTDs of COMMD4 and COMMD5 and was used as a starting point to build the rest of the CCDC93 model (**Fig. 3i**). Through manual building and placement of high confidence prediction segments from AF2, we traced the CCDC93 model to the I-coil region of the map (residues 1–313; **Fig. 3i**). We then used the same approach to build the middle region of CCDC22 (residues 109–322; **Fig. 3i**). Finally, we

validated the manually built parts of our model by comparing it to a model predicted by AF2 for this part of the complex (**Extended Data Fig. 3a**).

Finally, we built atomic models into the cryo-EM density of the bottom half of the Commander complex. The bottom half exhibits much more flexibility than the top half, as evidenced by the lower overall resolution of the focused maps (**Fig. 2c-d**). AF2 predictions, although predicting the overall arrangement mostly correct, failed to reproduce a single model that would directly fit the density in this region. Therefore, we generated the initial model in several parts and fitted them individually in the cryo-EM density before merging them to generate a complete model of this region (**Extended Data Fig. 4a-c**).

COMMD-ring is highly interconnected and stable

Our AP-MS results suggest that the COMMD proteins form a stable complex containing all of the ten proteins. Interestingly, the COMMD proteins are observed to form two separating clusters, with COMMD5, 6, 2, 9, and 10 in one and COMMD1, 8, 3, and 4 in the other, possibly corresponding to two regions within the structure. In our final atomic model of the Commander complex, the COMMD-ring exhibits pseudo-D5 symmetry and is characterized by four types of pairwise interactions around the ring. We denote these in order of descending buried interface area as handshake (HS), wristbump (WB), over-1, and over-2 interactions (**Fig. 3c-h, Extended Data Fig. 3c-d**). HS interactions have been described in the literature, and constitute a large, buried surface area (1958-2723 $Å^2$) between two COMMD domains (**Fig. 3e**). This surface is mediated by the C-terminal helix (CH) and the three-stranded β-sheet of the COMMD fold clasping the opposing COMMD domain in a pseudo-C2-symmetric interaction. HS interactions are mainly mediated by sidechains. The WB interaction, in contrast, is defined by a much smaller interface (554-620 $Å^2$) and consists of a highly curved intermolecular β-sheet, formed between the β1 of each COMMD. WBs are mainly mediated by backbone interactions that span from the conserved Trp on one COMMD to the respective Trp on the other COMMD (**Extended Data Fig. 3b**). Both HS and WB interactions take place between COMMD subunits with NTDs pointing in opposite directions relative to the COMMD-ring (**Fig. 3c**). The remaining two interfaces, over-1 and over-2, are smaller (<500 $Å^2$). Over-1 interactions occur between COMMDs with NTDs on the same side of the ring and localize on the linker between the COMMD domain and the NTD, the β2–β3 loop contacting the β-sheet of the opposing COMMD subunit, and the CH contacting the β1–β2 loop (**Fig. 3g**). Only one significant over-2 interface exists between COMMD2 and COMMD5 (456 $Å^2$), which is due to the kink in the CH of COMMD2. This in turn allows interactions between the CHs in addition to the interactions between CH of COMMD2 and the NTD of COMMD5 (**Fig. 3h**). These pairwise interactions together constitute a tightly bound core structure with several complementary interaction surfaces from one COMMD protein to up to five neighboring COMMD proteins. The NTDs of COMMDs

1, 7, 9 and 10 are less ordered than the rest. This is likely due to the flexibility of the linker between the domains. In addition, the NTDs of these COMMDs have the least stabilizing interactions with the CCDCs, with COMMD1 and COMMD9 having none, and COMMD7 and COMMD10 contacting only short helical segments of CCDC22 situated between flexible regions (**Extended Data Fig. 3e**).

PTMs

As interactions of the Commander complex might be PTM regulated, we searched for possible post translational modifications (PTMs) from the AP-MS data. This analysis identified several phosphorylation sites in six of the complex components, and histidine methylation in five components. (**Supplementary Data 2**; **Extended Data Fig. 5a**).
