# Supplementary material for: Structure and interactions of the endogenous human Commander complex
Source: Nat Struct Mol Biol. 2024 Mar 8;31(6):925–38. doi: 10.1038/s41594-024-01246-1 (PMC11189303; doi:10.1038/s41594-024-01246-1)
Supplement: Supplementary file 2 — Reporting Summary [file 41594_2024_1246_MOESM2_ESM.pdf]

nature portfolio

Corresponding author(s): Markku Varjosalo

Last updated by author(s): Dec 20, 2023

Reporting Summary

Nature Portfolio wishes to improve the reproducibility of the work that we publish. This form provides structure for consistency and transparency in reporting. For further information on Nature Portfolio policies, see our Editorial Policies and the Editorial Policy Checklist.

Statistics

For all statistical analyses, confirm that the following items are present in the figure legend, table legend, main text, or Methods section.

n/a	Confirmed	
☐	☒	The exact sample size (*n*) for each experimental group/condition, given as a discrete number and unit of measurement
☐	☒	A statement on whether measurements were taken from distinct samples or whether the same sample was measured repeatedly
☒	☐	The statistical test(s) used AND whether they are one- or two-sided<br>*Only common tests should be described solely by name; describe more complex techniques in the Methods section.*
☒	☐	A description of all covariates tested
☒	☐	A description of any assumptions or corrections, such as tests of normality and adjustment for multiple comparisons
☐	☒	A full description of the statistical parameters including central tendency (e.g. means) or other basic estimates (e.g. regression coefficient) AND variation (e.g. standard deviation) or associated estimates of uncertainty (e.g. confidence intervals)
☒	☐	For null hypothesis testing, the test statistic (e.g. *F*, *t*, *r*) with confidence intervals, effect sizes, degrees of freedom and *P* value noted<br>*Give P values as exact values whenever suitable.*
☒	☐	For Bayesian analysis, information on the choice of priors and Markov chain Monte Carlo settings
☒	☐	For hierarchical and complex designs, identification of the appropriate level for tests and full reporting of outcomes
☒	☐	Estimates of effect sizes (e.g. Cohen's *d*, Pearson's *r*), indicating how they were calculated

Our web collection on statistics for biologists contains articles on many of the points above.

Software and code

Policy information about availability of computer code

Data collection

CryoEM data was collected using EPU software (version 2.11.0, Thermo-Fisher). The MS data was collected in the study using commercial Bruker Daltonics timsControl (version 4.1.12) & HyStar (version 6.2.1.13) software.

Data analysis

CryoEM data was analysed principally using cryoSPARC (v. 4.1.1), with additional particle picking routines performed using TOPAZ (version 0.2.4). Initial data processing was carried out using implementations of RELION (v. 3.1) and crYOLO (v. 1.8.0) within the Scipion framework and utilities therein.
Maps and models were analyzed using ChimeraX (v. 1.5), and models built using a combination of ISOLDE (v. 1.5) and PHENIX (version 1.21).
Initial models were obtained by prediction using AlphaFold v. 2.3.1 and AlphaFold-Multimer (v3) via local installation of ColabFold (v. 1.5.2).

CRAPome, Mellacheruvu et al., 2013 http://www.crapome.org/
Cytoscape version 3.1.0, Shannon et al., 2003 http://www.cytoscape.org/
DAVID Bioinformatics Resources, National Institute of Allergy and Infectious Diseases (NIAID), NIH https://david.ncifcrf.gov/home.jsp
Gene Ontology analysis tool DAVID, Bioinformatics Resources 6.8 National Institute of Allergy and Infectious Diseases (NIAID), NIH https://david.ncifcrf.gov/home.jsp
Hierarchial clustering tool Biohit-viz and Pro-Hits-viz, Gigras lab, Department of Molecular Genetics, University of Toronto, Toronto, Ontario, Canada https://prohits-viz.lunenfeld.ca
Interaction network analysis tool: PINA v2.0, Cowley et al., 2012 http://cbg.garvan.unsw.edu.au/pina/
SAINTexpress version 3.1.0, Choi et al., 2011, http://saint-apms.sourceforge.net/Main.html

For manuscripts utilizing custom algorithms or software that are central to the research but not yet described in published literature, software must be made available to editors and reviewers. We strongly encourage code deposition in a community repository (e.g. GitHub). See the Nature Portfolio guidelines for submitting code & software for further information.

Data

Policy information about availability of data

All manuscripts must include a data availability statement. This statement should provide the following information, where applicable:

- Accession codes, unique identifiers, or web links for publicly available datasets
- A description of any restrictions on data availability
- For clinical datasets or third party data, please ensure that the statement adheres to our policy

Databases used in the study:
Mammalian protein complex resource: CORUM Institute of Bioinformatics and Systems Biology, Helmholtz Zentrum München, http://mips.helmholtz-muenchen.de/corum/
Uniprot: https://www.uniprot.org/

Electron microscopy maps are deposited in the electron microscopy data bank (EMDB) with accession codes EMD-17340, EMD-17339, EMD-17341 and EMD-17342. Protein structure models were deposited into the protein data bank (PDB) with accession codes 8POW, 8POV and 8POX.

The source data of the figures are provided with this paper as a separate Excel sheet. The MS peptide raw data from the MS runs have been deposited in the Massive database (https://massive.ucsd.edu/ProteoSAFe/private-dataset.jsp?task=985dbde9f67146758bbd959b3847703f) under accession number MSV000091490

Material Availability
Plasmids generated in this study will be deposited in Addgene. No other unique reagents were generated in this study.

Research involving human participants, their data, or biological material

Policy information about studies with human participants or human data. See also policy information about sex, gender (identity/presentation), and sexual orientation and race, ethnicity and racism.

Reporting on sex and gender	N/A
Reporting on race, ethnicity, or other socially relevant groupings	N/A
Population characteristics	N/A
Recruitment	N/A
Ethics oversight	N/A

Note that full information on the approval of the study protocol must also be provided in the manuscript.

Field-specific reporting

Please select the one below that is the best fit for your research. If you are not sure, read the appropriate sections before making your selection.

- [x] Life sciences
- [ ] Behavioural & social sciences
- [ ] Ecological, evolutionary & environmental sciences

For a reference copy of the document with all sections, see nature.com/documents/nr-reporting-summary-flat.pdf

Life sciences study design

All studies must disclose on these points even when the disclosure is negative.

Sample size	Sufficient sample size (number of cryoEM images and extracted particles) was determined by collecting enough data that would result a reconstructed map at sufficient resolution (estimated by Fourier shell correlation). For other experiments sufficient sample sizes were chosen for each experiment to determine whether the outcome was statistically significant. For cell line experiments minimal number replicates for each experiments was two or more
Data exclusions	Micrographs that failed in contrast transfer function estimation were discarded. Particles that did not show features of the complex were discarded in 2D-classification step.
Replication	The data were divided in two random subsets and processed separately as part of CryoSPARC's gold standard refinement protocol. All attempts at replication were successful. When applicable, experiments were independently repeated minimally twice.
Randomization	CryoEM data were randomly split in two half data sets in the beginning of the image processing (gold standard refinement). Other type of

Randomization	randomisation is not relevant for cryoEM as data collected represents unbiased views of the sample. Different samples (different baits or different analysis method) were allocated to MS analysis in a random order
Blinding	Investigators were not blinded for any of the analyses as the analytical methods employed were automated and do not allow for subjective interpretation.

Reporting for specific materials, systems and methods

We require information from authors about some types of materials, experimental systems and methods used in many studies. Here, indicate whether each material, system or method listed is relevant to your study. If you are not sure if a list item applies to your research, read the appropriate section before selecting a response.

Materials & experimental systems

n/a	Involved in the study
☒	☐ Antibodies
☐	☒ Eukaryotic cell lines
☒	☐ Palaeontology and archaeology
☒	☐ Animals and other organisms
☒	☐ Clinical data
☒	☐ Dual use research of concern
☒	☐ Plants

Methods

n/a	Involved in the study
☒	☐ ChIP-seq
☒	☐ Flow cytometry
☒	☐ MRI-based neuroimaging

Eukaryotic cell lines

Policy information about cell lines and Sex and Gender in Research

Cell line source(s)	HEK293 Flp-In T-REx (Thermo-Fisher)
Authentication	The cell line was obtained directly from commercial sources or biological repositories; additionally only low passage cells (passage number <10) were used for experiments.
Mycoplasma contamination	cell lines tested negative for mycoplasma
Commonly misidentified lines (See ICLAC register)	no commonly misidentified cell lines used
